# *OsMOCSalt* as a candidate salt tolerance gene in rice (*Oryza sativa*) identified through meta-analysis, overview analysis and collinearity analysis

**DOI:** 10.3389/fpls.2026.1868272

**Published:** 2026-07-24

**Authors:** Xiannan Zeng, Longji Chen, Xianya Dong, Tianning Zhuang, Runqing Liu, Yitong Yin, Zilong Tang, Ziqi Zhu, Conghe Liu, Qiulai Song, Shaokun Zhang, Sun Yu, Di Cao, Jinghan Fang, Quanxi Liang, Qi Wang, Qi Zhang

**Affiliations:** 1 Institute of Crop Cultivation and Tillage, Heilongjiang Academy of Agricultural Sciences, Harbin, China; 2 Agricultural College Heilongjiang Bayi Agricultural Reclamation University Daqing Heilongjiang, National Cereals Technology Engineering Research Center, Daqing, China; 3 Heilongjiang Vocational University of Agricultural Engineering, Harbin, China

**Keywords:** meta, overview, collinearity analysis, molecular markers, rice (*Oryza sativa*), salt tolerance, verification

## Abstract

Salinization poses a critical threat to global crop production, affecting approximately 15% of irrigated land and substantially reducing yields. Therefore, enhancing crop salt tolerance has become extremely urgent. Notably, identifying and utilizing salt-related genes is fundamental to salt tolerance breeding. Rice (*Oryza sativa*) is an important crop and a staple food worldwide, especially in Asia. However, most cultivated varieties are sensitive to salt stress. Therefore, identifying and applying salt-related genes could facilitate breeding of salt-tolerant varieties. In this study, 265 salt-related QTLs were obtained from the Gramene database and subjected to overview and collinearity analyses. Notably, the QTLs were concentrated on chromosome (Chr) 9, within 12.79–15.29 Mb in the overview analysis and 14.80–18.68 Mb in the collinearity analysis. Additionally, 65 meta-QTLs (MQTLs) were identified through meta-analysis. The candidate interval for MQTL9.4 was 5.2 Mb. Integrating the three analyses, a candidate region associated with salt tolerance was defined on Chr 9, at 14.85–15.21 Mb. A total of 36 genes were identified within this region. Screening based on predicted molecular function, expression profiles across different tissues, and responses to salt stress revealed the candidate gene *Os09g0416200*, designated *OsMOCSalt*. This gene had two haplotypes, and *OsMOCSalt^Hap1^* was confirmed to positively regulate salt tolerance in transgenic yeast. Additionally, molecular markers associated with the polymorphic sites were developed, and a specific single nucleotide polymorphism was found to be significantly correlated with salt tolerance (*P < 0.05*). This study identified and validated a salt tolerance-related gene, providing an empirical basis for the molecular breeding of salt-tolerant rice.

## Graphical Abstract

**Figure:**
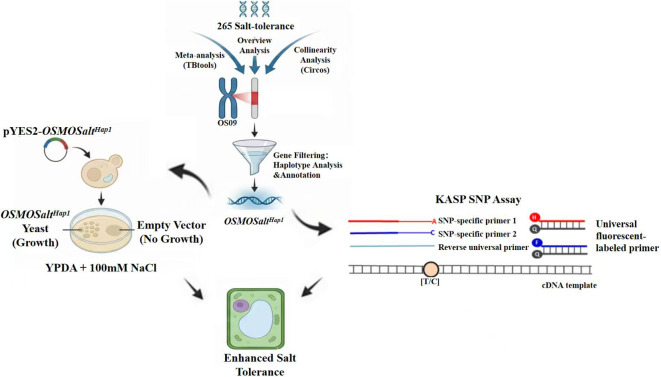

## 1 Introduction

Soil salinization has become an inescapable global challenge amid increasing population size and the deterioration of the natural environment, with approximately 7% of arable land affected by salt stress (Tian et al., 2025). Salt stress caused by soil salinization has emerged as a major factor limiting crop production globally (Che et al., 2024) through the excessive accumulation of salts (Zhou et al., 2025). The main damage caused by salt stress arises from high-concentration Na^+^ entering cells through ion channels and carrier proteins, as well as excessively high extracellular Na^+^ concentrations, which reduce cellular osmotic potential, causing water efflux from cells (Xiao et al., 2026). Salt stress adversely affects plants by inhibiting seed germination, growth and development, flowering, and grain formation (Park et al., 2013). High Na^+^ concentration in saline soil limits the absorption of water and nutrients by plants (Gong, 2021).

Water deficit and nutrient imbalance under salt stress are the main factors that hinder plant growth and development. Osmotic and ionic stresses further induce oxidative stress, resulting in reduced stomatal conductance, transpiration, photosynthesis, and growth (Morton et al., 2019). Salt stress limits photosynthesis, physiological metabolism, growth, and development, thereby severely affecting yield (Dong et al., 2025). Therefore, improving salt tolerance has practical importance for crop production. Notably, identifying salt tolerance genes and utilizing them in breeding programs is a direct approach to enhance salt tolerance (Han et al., 2025).

Advances in bioinformatics have led to the development of several analytical methods for mining and analyzing related genes using databases (Guan et al., 2023). Collinearity analysis characterizes the conservation of gene types and the order of their arrangement across species that have evolved from a common ancestor (McCouch, 2001). It thus reveals homologous relationships based on gene similarity and plays an important role in establishing gene associations (Coghlan et al., 2005). Target genes required for research can be identified using collinearity analysis because eukaryotic genomes across different species exhibit strong collinearity at multiple levels throughout evolution (Coghlan et al., 2005). Consequently, collinearity analysis has become an important approach in studying biological evolution and is a standard method for elucidating the evolutionary history of genomes and genes (Xu et al., 2016).

Overview analysis is a method based on statistical principles that systematically integrates genotype information and related data (Shaw, 2008), eliminates redundant information, clarifies core research targets, narrows the genomic regions associated with key traits, and identifies candidate intervals for important candidate genes (Wang et al., 2016). Additionally, meta-analysis is a quantitative method initially applied in medicine to systematically combine and comprehensively analyze multiple independent studies with the same objective (Glass, 1976). It is currently widely applied in many scientific fields, including genetic breeding (Chardon et al., 2004).

For instance, rice flowering genes have been mapped to 19 QTLs through these approaches (Chardon et al., 2004). In another study, analysis of 62 QTLs associated with resistance to soybean cyst nematode identified two important candidate genes (Guo et al., 2006). In addition, 11 QTLs related to oil content were mapped using soybean populations, and database QTLs were subsequently integrated based on previously reported results (Qi et al., 2011). Meta-analysis was subsequently conducted to identify candidate genes and develop corresponding molecular markers. Numerous genes, including soybean protein genes (Gong et al., 2018) and drought tolerance genes in maize (Hao et al., 2010), have been discovered and applied using these approaches. These studies demonstrate the effectiveness of integrative QTL approaches in identifying robust candidate genes.

Rice (*Oryza sativa*) is a major global food crop and a staple for more than half of the world’s population (Talarico et al., 2025). Notably, rice is highly sensitive to salt stress, which significantly constrains its yield. Salt stress causes plant wilting and directly reduces yields (Li et al., 2024). High-quality farmland suitable for rice cultivation continues to decline owing to intensifying climate change and the expansion of saline land (Saleem et al., 2025). Therefore, research on salt-tolerant rice breeding has crucial strategic importance and practical value (Tiwari et al., 2024) and remains a key and urgent task in rice breeding (Pagnotta, 2025).

In this study, 265 salt-related QTLs were obtained from the Gramene database and subsequently subjected to overview analysis, collinearity analysis, and meta-analysis to localize candidate genes. Thus, a candidate gene was identified and subsequently validated to confer salt tolerance through gene annotation, functional prediction, and expression analysis. Additionally, Kompetitive Allele Specific PCR (KASP) molecular markers were developed and applied to germplasm screening, further providing a basis for genetic analysis and salt tolerance breeding.

## 2 Materials and methods

### 2.1 Meta-analysis, overview analysis, and collinearity analysis

Quantitative trait loci (QTL) with detailed information on salt tolerance were collected from the Gramene database (https://www.gramene.org/). Notably, all the QTLs were published in peer-reviewed articles on rice salt tolerance published between 2009 and 2024. The physical map of rice was obtained from the Phytozome database (https://phytozome-next.jgi.doe.gov/) (Sosnowski et al., 2012). QTLs were retained when they were significantly associated with salt tolerance under control (N) or salt-stress (T) conditions, had clearly defined chromosomal positions, a logarithm of odds (LOD) score ≥ 2.0, and reported either phenotypic variance explained (*R*^2^) and confidence intervals (CIs) or sufficient data for CI estimation. Ambiguous, duplicated, low-LOD, or incomplete records were excluded (**Supplementary Table 1**) (Goffinet and Gerber, 2000).

For QTLs lacking a reported 95% CI, the interval was estimated using the empirical formula (Darvasi and Soller, 1997):


$$CI=\;163/\left(R^{2}\times \;N\right)$$

Here, CI represents the 95% confidence interval of a given QTL in centimorgans (cM), and *R*^2^ refers to the percentage of phenotypic variance explained by the corresponding QTL.

Multiple statistical analyses were performed to evaluate heterogeneity of the integrated QTL dataset to ensure the accuracy and reliability of the M-QTL identification results.

Heterogeneity among studies was evaluated using Cochran’s Q test and the I^2^ statistic. A random-effects model was applied when I^2^ exceeded 50%; otherwise, a fixed-effects model was used. Publication bias was assessed using funnel plots and Egger’s regression test, and the robustness of identified MQTLs was examined by leave-one-out sensitivity analysis. Subgroup analyses were conducted according to trait category, rice developmental stage, and mapping population type to explore potential sources of heterogeneity (Qin et al., 2018).

Overview analysis was used to refine consensus QTL intervals and estimate the probability of QTL occurrence along each chromosome. Each projected QTL was modeled as a normal distribution based on its position and variance, and the QTL density function was calculated at 0.5-cM intervals as follows:


$$p(x,x+\;0.5)=\frac{\sum_{i=1}^{nbqtl}\int_{x}^{x+0.5}N(Pi,Si^{2})d(x)}{nbE}$$

The uniformity likelihood function U(x) was used to represent the expected QTL occurrence per unit chromosome length, and H(x) was defined as five times U(x):


$$\text{U}(\text{x})=\frac{nbQTL/nbE}{Total\;length\;of\;map}\times 0.5;\text{ H}(\text{x})=\frac{nbQTL/nbE}{Total\;length\;of\;map}\times 0.5\times 5.$$

Here, nbQTL is the number of QTLs, nbE is the number of independent experiments, and Total length of map is the length of the reference genome map. Density peaks exceeding both U(x) and H(x) were regarded as likely true QTL positions (Yang et al., 2025).

Collinearity relationships among rice salt tolerance QTL intervals were analyzed using MCScanX and custom Perl scripts, and the resulting networks were visualized with Circos and Cytoscape (Zhang et al., 2022b; Tajo et al., 2023; Lu et al., 2025). Candidate regions were then refined by integrating the results of meta-analysis, overview analysis, and collinearity analysis. Genes within the overlapping consensus intervals were retrieved from Phytozome and functionally annotated using Phytozome to identify candidates potentially involved in salt tolerance.

The candidate range was further narrowed down by integrating the results of meta-analysis, overview analysis, and collinearity analysis. Genes within the consensus QTL interval were predicted using genomic information from the Phytozome database after Venn analysis of the three methods.

### 2.2 Genotype source and selection

Single nucleotide polymorphisms (SNPs) in the candidate genes were obtained from the Rice SNP-Seek Database and validated using whole-genome resequencing data from 50 representative accessions in our germplasm collection.

Allele frequencies of individual SNPs were compared using the chi-square test:


$${\text{χ}}^{2}=\sum \frac{{\left(\text{O}-\text{E}\right)}^{2}}{\text{E}}$$

Here, O and E are the observed and expected frequencies, respectively, under the null hypothesis of no association between genotype and phenotype.

Phenotypic differences in relative salt-alkali damage rate between genotype groups were evaluated using independent-samples *t*-tests, and effect size was estimated using Cohen’s d:


$$\text{d}=\frac{{\text{μ}}_{1}-{\text{μ}}_{2}}{\sqrt[{2}]{\frac{({\text{n}}_{1}-1){\text{s}}_{1}^{2}+({\text{n}}_{2}-1){\text{s}}_{2}^{2}}{{\text{n}}_{1}+{\text{n}}_{2}-2}}}$$

Here, μ, s, and n represent the mean, standard deviation, and sample size of each genotype group, respectively.

Haplotypes were inferred using the expectation-maximization algorithm in Haploview v4.2, and their associations with salt tolerance were evaluated by logistic regression, with salt tolerance status as the dependent variable and haplotype as the independent variable.

### 2.3 Validation of salt tolerance candidate genes and molecular marker development

Rice germplasm resources, including cultivated varieties, such as ‘Longjing 203’ and ‘Longjing 209’ from Northeast China, were obtained from the rice germplasm resource of Heilongjiang Academy of Agricultural Sciences for analysis (**Supplementary Table 2**). Identification of salt-tolerant germplasm resources had been previously conducted under laboratory conditions. The seeds that were selected as experimental materials were characterized by their plump grains, uniform size, and lack of disease or pest symptoms, while each group was set up with three biological replicates, with 30 seeds per replicate. First, the seeds were rinsed with clean water three to five times and then surface-sterilized with 75% anhydrous ethanol for 30 s. After promptly discarding the ethanol, the seeds were rinsed six to eight times with sterile ultrapure water. Finally, the washed seeds were placed on sterile filter paper to blot off surface moisture for later use. Germination and culture were carried out using the filter paper germination method. A germination chamber was used, and two layers of qualitative filter were laid at the bottom. The pretreated rice seeds were evenly placed on the filter paper, with equal spacing between seeds in order to avoid competition that could affect water uptake and germination. An appropriate amount of ultrapure water was added until the filter paper was completely moistened and no standing water remained at the bottom. The dishes containing the seeds were then placed in an intelligent artificial climate incubator to ensure constant-temperature, dark cultivation at 28°C and a relative humidity of 75–80%. During cultivation, the filter paper was maintained moist at all times, and the seeds were thus cultured for 10 days. The germination rate was calculated after 10 days of incubation, followed by the removal of rice seeds from the germination chamber. Seed germination was evaluated, and a seed was considered germinated when its radicle had reached the prescribed germination length and the shoot length was at least one-half of the grain length. The germination rate was calculated using the following formula, with three biological replicates per treatment:


$$Germination\;rate\;of\;rice\;seeds\;(\%)=\frac{Number\;of\;germinated\;seeds}{Total\;seeds}\times 100\%$$

The saline concentration was set at 125 mmol·L^-1^. The determination of the relative saline damage rate and classification of salt tolerance at the rice germination stage of rice varieties were conducted according to the evaluation criteria in **Supplementary Table 3**, and the formula for calculating the saline damage rate is as follows:


$$Relative\;salt\;damage\;rate\;(\%)=\frac{Control\;seed\;germination\;rate-Seed\;germination\;rate\;of\;test}{Germination\;rate\;of\;control\;seed}$$

The specific classification standard for saline resistance during germination is outlined in **Supplementary Table 4**.

DNA and RNA from the samples were extracted from plant root tissue using commercial kits under control and salt-treatment conditions. DNA and RNA quality were assessed by 1.5% agarose gel electrophoresis and Nanodrop analysis (Nanodrop Technologies, Wilmington, DE, USA). Qualified samples were used for subsequent analysis.

Expression of candidate genes was analyzed by RT-qPCR and quantified using the 2^–ΔCt^ method, with biological and technical replicates. In contrast, |log_2_(fold change)| between salt-treated and control samples was determined in biological replicates. RT-qPCR primers were designed using Primer 5.0. *OsActin* was used as the reference gene (**Supplementary Table 5**).

The coding sequence (CDS) of the candidate gene (*OsMOCSalt*) was cloned into the pYes2 vector, and PEG-mediated transformation was used to introduce pYes2-*OsMOCSalt* into the yeast strain *INVScI* (primers are listed in **Supplementary Table 6**). The transformed strains were inoculated onto normal YPDA solid medium and YPDA medium containing NaCl in order to verify *OsMOCSalt* function under salt stress.

KASP primers were designed for SNP sites within the candidate gene. SnapGene software was used to design KASP molecular markers, and ULTRAPAGE purification was applied. The KASP primers are listed in **Supplementary Table 7**.

## 3 Results

### 3.1 Overview analysis and collinearity analysis of QTLs

**Figure 1:**
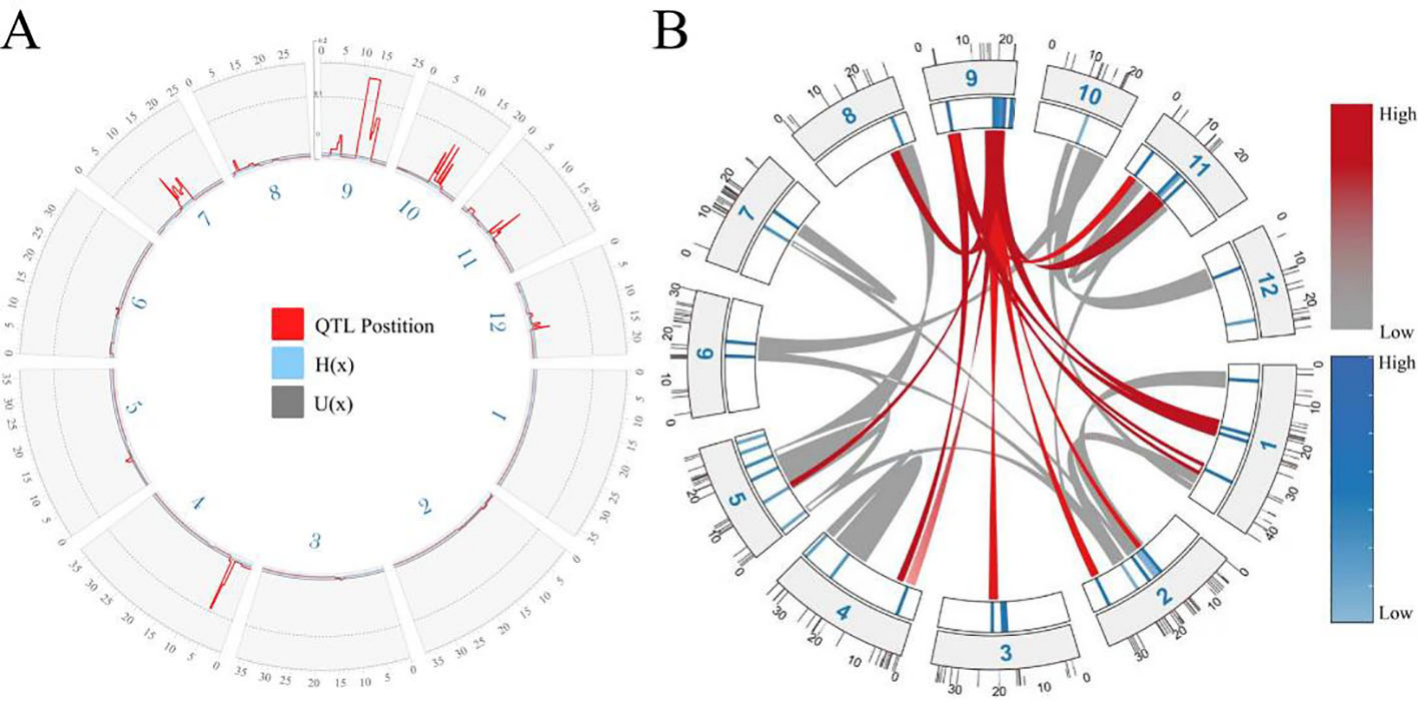
Overview and collinearity analysis of salt tolerance-related QTLs. **(A)** Overview analysis of salt tolerance-related QTLs. Overview curves of QTL distributions across chromosomes are shown, with the vertical axis representing frequency. **(B)** Collinearity analysis of salt tolerance-related QTLs. Lines within the circle, ranging from gray to red, represent collinear relationships, while blue boxes indicate gene density across chromosomes.

A total of 265 QTLs related to salt tolerance were included in the overview analysis, with all 12 chromosomes containing QTLS, though the frequencies varied. Only QTLs with a value greater than 0.1 were considered, and the effective QTL region spanned from 12.79 to 15.29 Mb on Os09 (**Figure 1A**), indicating that this region was highly associated with rice salt tolerance. Additionally, QTL information for salt tolerance was subjected to collinearity analysis, and the results were visualized using Circos software (**Figure 1B**). The central QTL showing the highest collinearity with other QTLs was located on Os09. By comparing the results of the collinearity module analysis, the most significantly enriched interval was identified between 14.80 and 17.68 Mb.

### 3.2 Meta-analysis of QTLs

**Figure 2:**
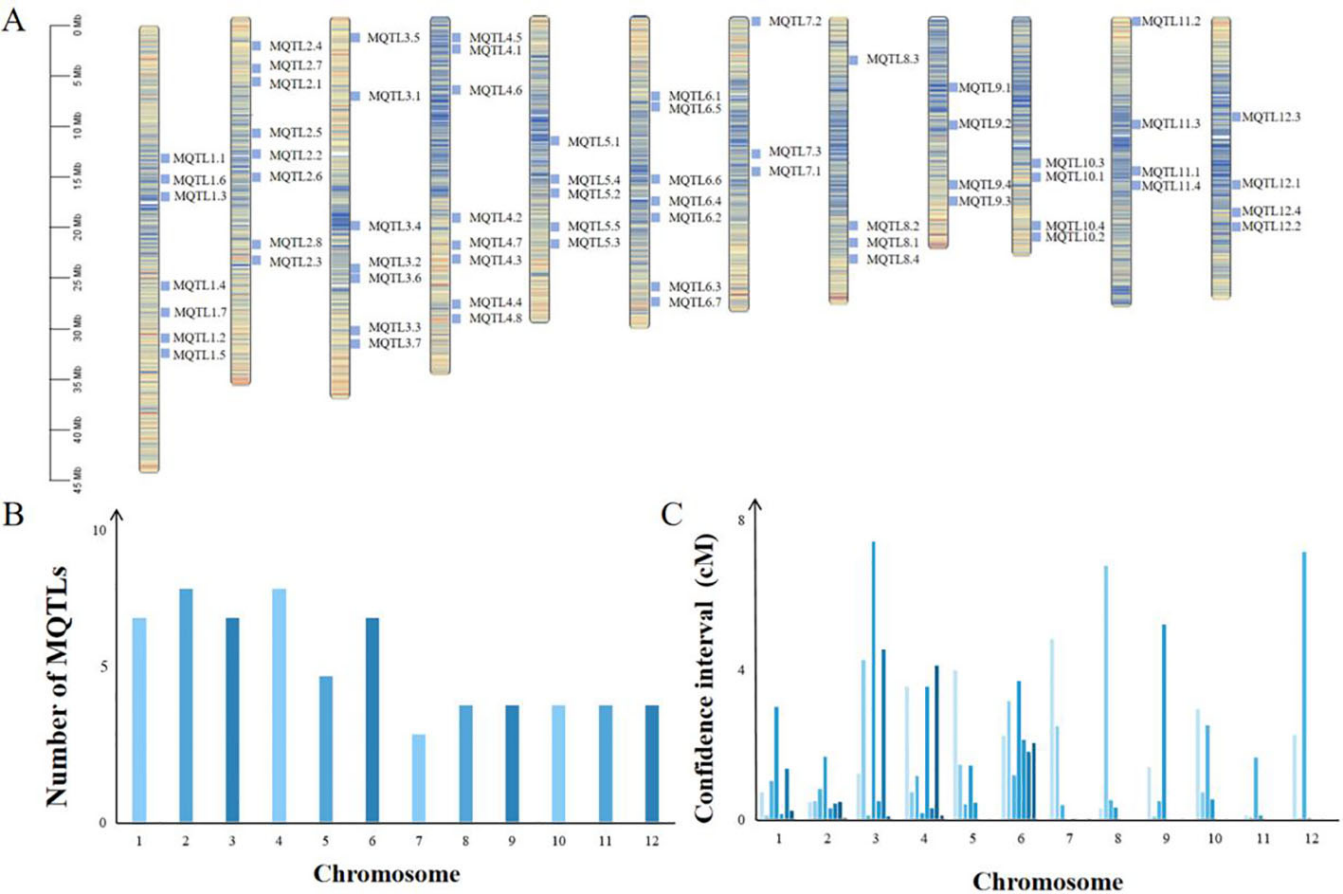
Meta-analysis of salt tolerance-related QTLs. **(A)** Distribution of MQTLs across rice chromosomes; blue squares indicate MQTL positions, while the gradient from blue to red represents gene density from low to high. **(B)** The number of MQTLs identified on each chromosome. **(C)** Confidence interval of each MQTL across chromosomes.

A total of 265 original QTLs were mapped by meta-QTL analysis, such that more regions with a higher number of overlapping QTLs were considered more reliable meta-QTLs (MQTLs). These selected MQTL regions exhibited strong statistical significance based on a comprehensive evaluation of the association between genes and salt tolerance traits, genotype frequency distributions, and genomic localization information. Among these MQTLs, on chromosome 9, the 95% confidence interval of MQTL9.4 was 14.85 to 15.21Mb, which represents a reliable candidate interval (**Figure 2**).

### 3.3 Venn analysis of overview analysis, collinearity analysis, and meta-analysis

**Figure 3:**
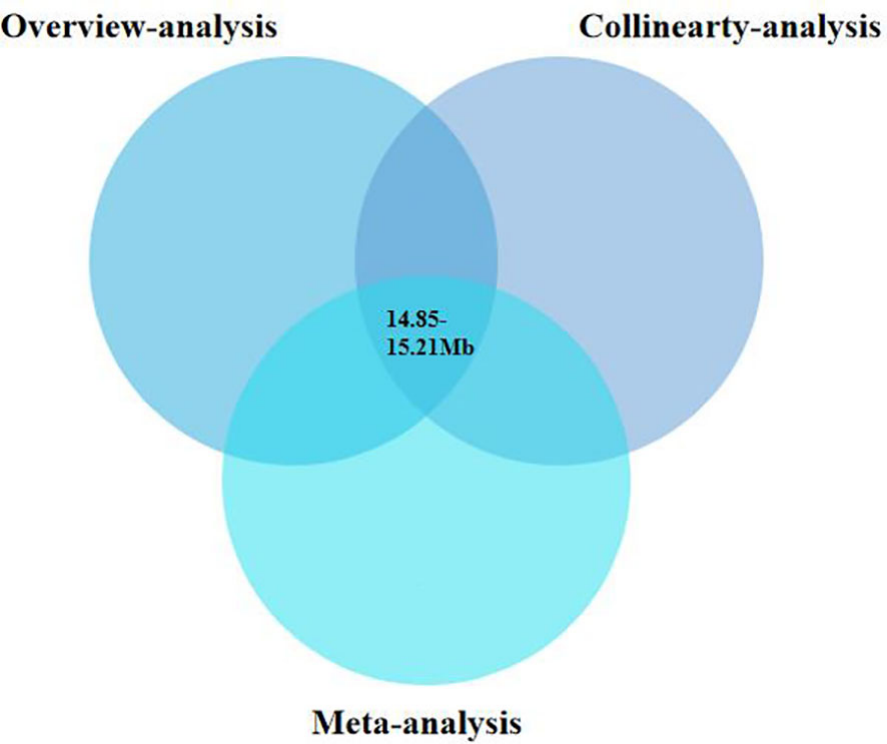
Venn of overview, collinearity, and meta-analysis. The overlapping region among the three circles represents the candidate interval on chromosome 9.

The results from all analyses (including overview analysis, collinearity analysis, and meta-analysis) identified an overlapping QTL region on chromosome 9 concentrated between 14.85 and 15.21 Mb. This intersection region can be considered an important candidate interval for one or more genes associated with salt tolerance (**Figure 3**).

### 3.4 Functional annotation and expression analysis

**Figure 4:**
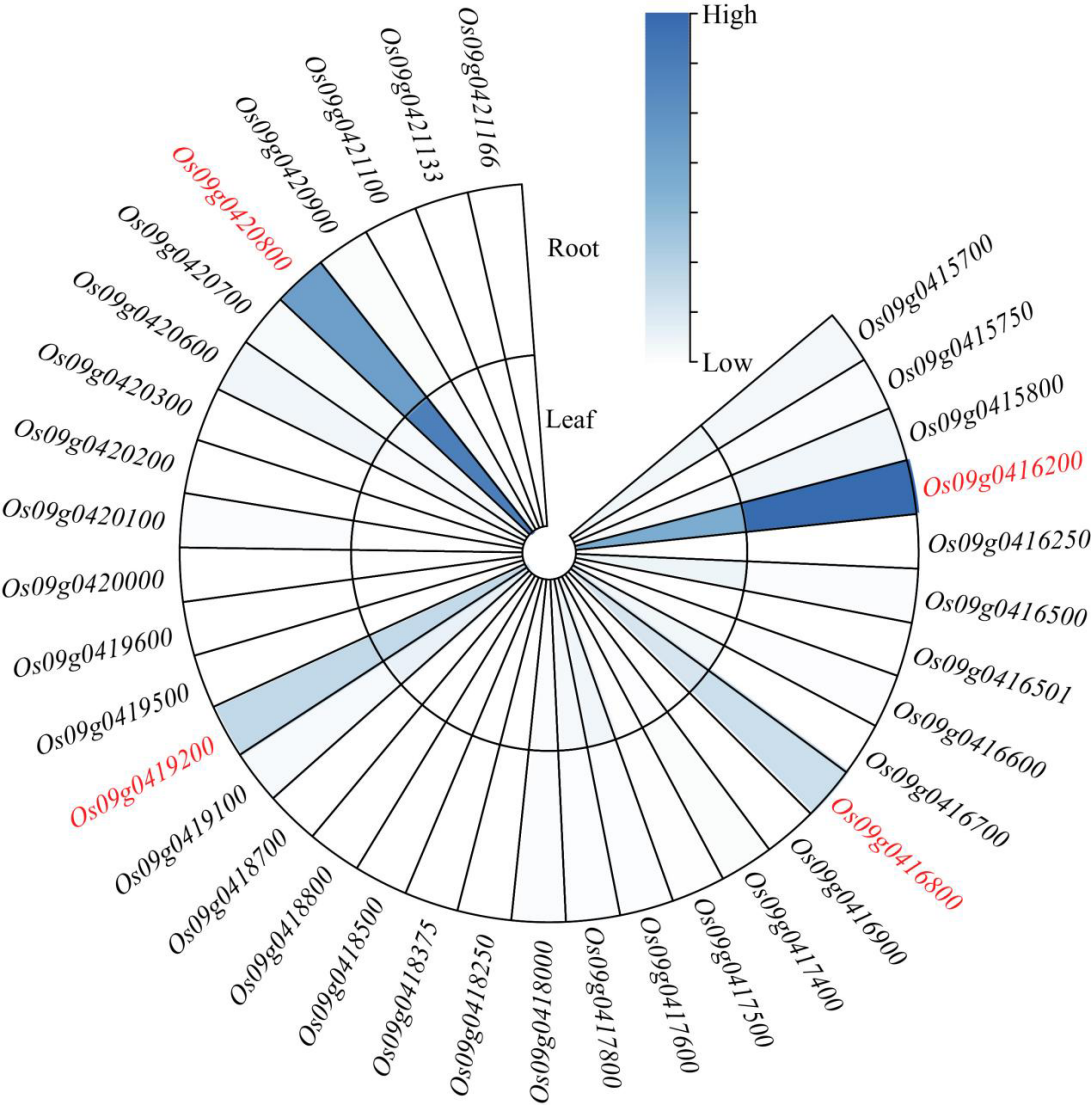
Heatmap of 36 genes within the candidate region on chromosome 9. The color scale from white to blue indicates gene expression levels from low to high, respectively.

Thirty-six genes were annotated within this region. Additionally, the expression data were obtained from public databases, and a heatmap was generated for these genes. Notably, 16 genes had no detectable expression. In addition, the expression levels of another 16 genes were very low (<1), suggesting that their reduced expression may not have a substantial functional impact. Only four genes (*Os09g0416200*, *Os09g0416800*, *Os09g0419200*, and *Os09g0420800*) exhibited relatively high expression levels (shown in red in **Figure 4**), which may serve as candidate genes for further analysis.

### 3.5 Expression response to salt stress

**Figure 5:**
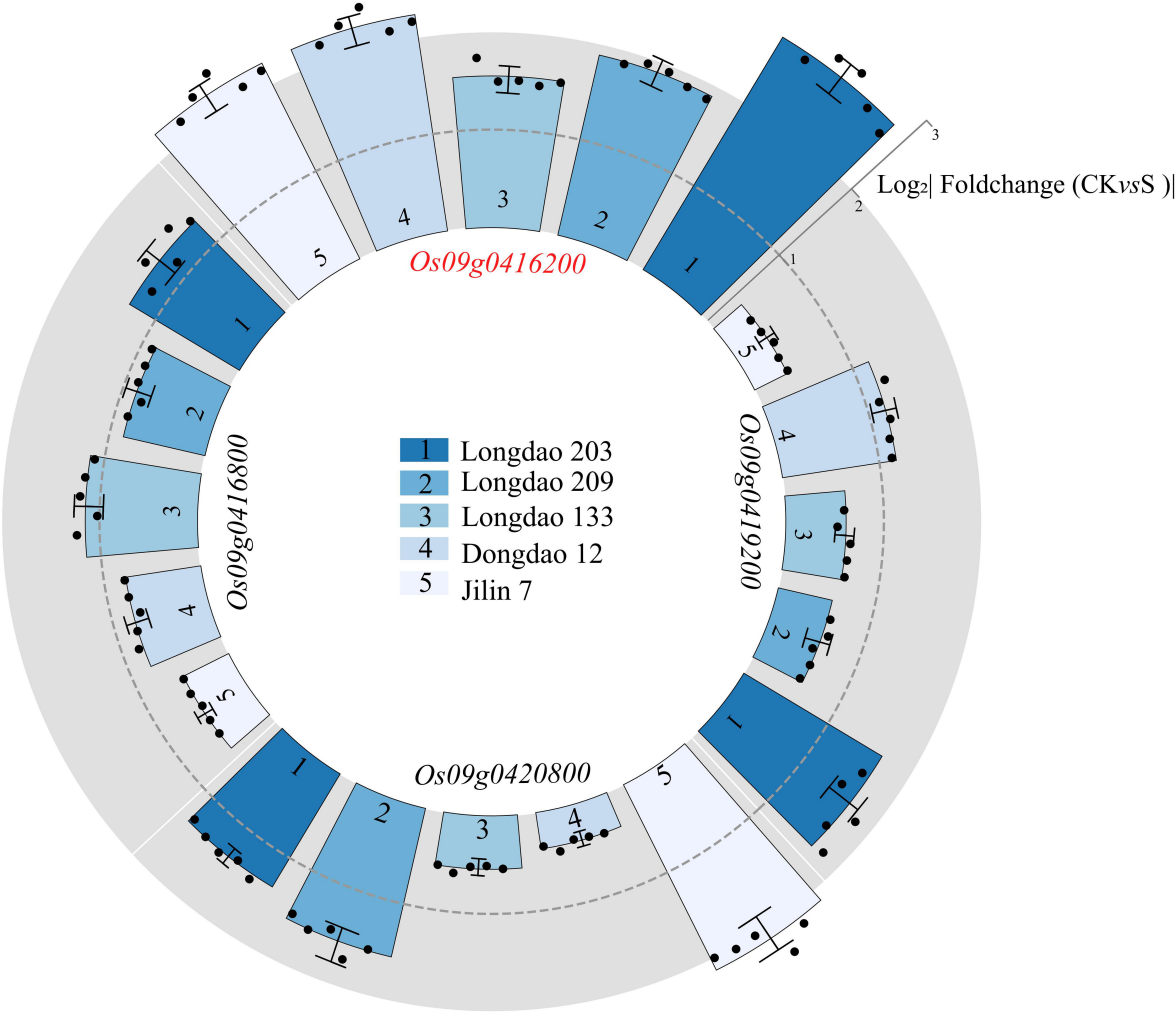
Expression analysis of four candidate genes under control (CK) and salt stress (S) conditions. Gene names within the circles represent the four candidate genes, while the vertical axis indicates the values of |log_2_Fold change (CK *vs*. S)|.

Five salt-tolerant cultivars, including ‘Longdao 203’ (Cultivated 1), ‘Longdao 209’ (Cultivated 2), ‘Longdao 133’ (Cultivated 3), ‘Dongdao 12’ (Cultivated 4), and ‘Jilin 7’ (Cultivated 5), were used to evaluate gene expression under salt stress. Differences in expression between control and salt-stress conditions, captured as |log_2_Fold change (CK *vs*. S)| values, were evaluated to identify candidate genes. Only *Os09g0416200* exhibited significant differences in expression levels between treatments. In contrast, the expression levels of other genes (*Os09g0416800*, *Os09g0419200*, and *Os09g0420800*) did not show consistent significant changes across all cultivars under stress conditions (**Figure 5**). These findings indicate that *Os09g0416200* is a strong potential candidate gene, and it was designated *OsMOCSalt*.

### 3.6 Analysis and verification of OsMOCSalt

**Figure 6:**
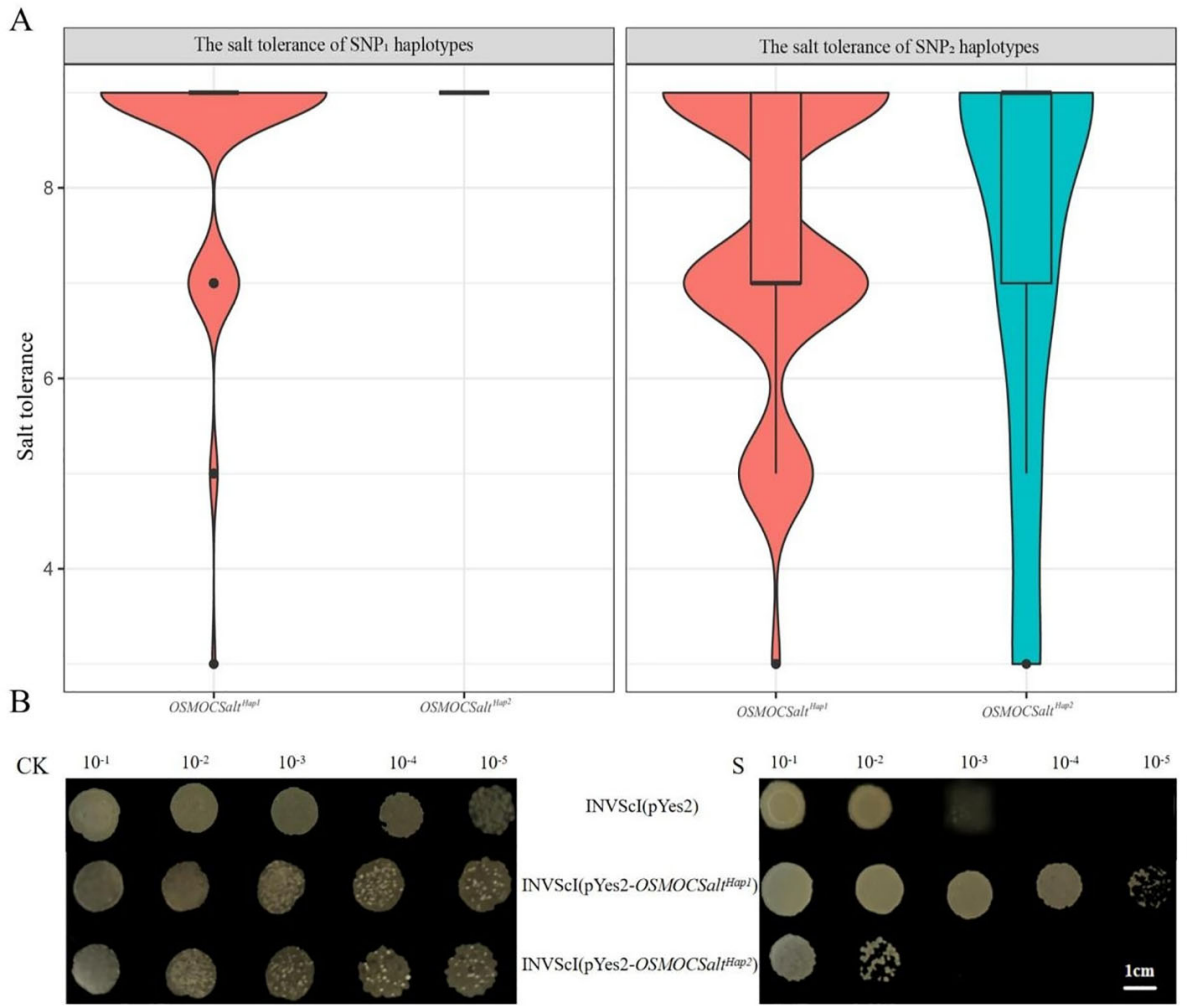
Analysis and functional verification of *OsMOCSalt.* **(A)** Haplotype analysis of *OsMOCSalt*; the left panel represents SNP_1_ sites, and the right panel represents SNP_2_ sites. **(B)** Growth analysis of transgenic yeast expressing different *OsMOCSalt* alleles under control and salt stress conditions.

SNP loci in the CDS region of the predicted gene *OsMOCSalt* were analyzed based on haplotype analysis from the database, and it was observed that two SNP loci occurred in the nucleotide sequence. These SNPs were located at positions 4487 (SNP_1_) and 6185 bp (SNP_2_), with T/C and A/G substitutions relative to the CDS sequence, respectively. The T allele of SNP_1_ in *OsMOCSalt* rice conferred higher salt tolerance than the C allele, while the two alleles of SNP_2_ showed no difference in salt tolerance (**Figure 6A**). Furthermore, transgenic yeast carrying different SNP_1_ alleles were used to validate the function of *OsMOCSalt* in this study. Transgenic pYES2-*OsMOCSalt^Hap1^* yeast exhibited significant differences in growth compared with pYES2-*OsMOCSalt^Hap2^* under salt stress in YPDA medium. In contrast, no difference in yeast growth was observed under normal YPDA conditions. These results indicate that transgenic pYES2-*OsMOCSalt^Hap1^* yeast exhibited enhanced salt tolerance, further demonstrating that *OsMOCSalt^Hap1^* is a favorable salt-tolerant allele of SNP_1_ (**Figure 6B**).

**Figure 7:**
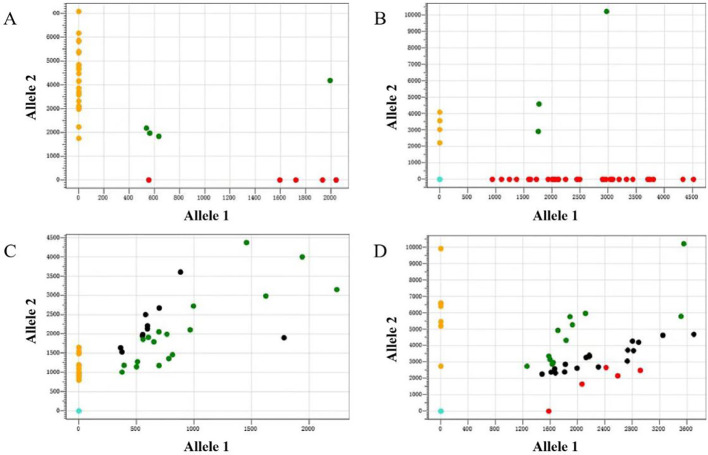
The molecular marker analysis of *OsMOCSalt.* **(A)** Genotyping analysis of SNP_1_ sites in salt-tolerant plants using KASP markers. **(B)** Genotyping analysis of SNP_1_ sites in salt-sensitive plants using KASP markers. **(C)** Genotyping analysis of SNP_2_ sites in salt-tolerant plants using KASP markers. **(D)** Genotyping analysis of SNP_2_ sites in salt-sensitive plants using KASP markers.

The molecular KASP markers for extreme salt tolerance materials in germplasm resources were also analyzed. SNP_1_ sites were more distinct in DNA genotyping between salt-tolerant and salt-sensitive plants. In salt-tolerant plants, the number of *OsMOCSalt^Hap1^* genotypes (T) was higher than that of *OsMOCSalt^Hap2^* (C), while in salt-sensitive plants, the number of *OsMOCSalt^Hap2^* (C) genotypes was higher than that of *OsMOCSalt^Hap1^* (T). However, the SNP_2_ genotype was not significantly associated with plant salt tolerance (**Figure 7**). These results suggest that SNP_1_ may influence salt tolerance, while *OsMOCSalt^Hap1^* represents the salt-tolerant haplotype.

## 4 Discussion

### 4.1 Identification of salt tolerance genes by integrative analysis

More than 100 QTLs for abiotic stress responses have been identified, but only a few have been verified as stable QTLs across different environments (Liang et al., 2018). Identification and screening of salt tolerance-related genes are important for enhancing ensuring adaptability to salinized soils and breeding salt-tolerant cultivars (Mirdar Mansuri et al., 2020). Salt tolerance-related QTLs may show significant variation among mapped QTLs, which may be owing to differences in experimental design, including genetic populations, progeny population sizes, and mapping methods, as well as differences among diverse population types such as RILs, CSSLs, F_2_, and BC populations (Shafiq et al., 2025). Although salt tolerance in rice is recognized as a typical stage-specific quantitative trait, the genetic loci and functional genes regulating salt responses vary substantially across phenological phases (Satasiya et al., 2024). Consequently, salt tolerance QTLs identified at the vegetative stage may not fully capture the genetic basis of salt adaptability at the reproductive stage, limiting the value of single-stage QTLs in molecular breeding. Notably, grouping QTLs according to growth stage in rice can help elucidate the genetic mechanism underlying stage-dependent salt tolerance and facilitate the identification of stage-specific MQTLs for targeted breeding. Nevertheless, integrating multi-stage QTL datasets via meta-analysis possesses unique merits and can improve the accuracy and practical value of core loci screening (Liu et al., 2024). The large-scale integration of heterogeneous datasets can reduce background noise caused by individual experiments, different genetic populations, and focus on single growth periods. Consequently, the consolidated MQTLs screened from extensive heterogeneous QTL resources are more likely to represent conserved, universal, stable, and generally important genomic regions associated with salt tolerance throughout the rice life cycle (Chandrasekaran et al., 2019). Therefore, this meta-analysis-based approach enables the identification of robust pleiotropic loci that function across multiple developmental stages, complementing single-stage QTL mapping and providing valuable genetic resources for breeding rice varieties with broad-spectrum salt tolerance throughout the growth cycle. Notably, some studies suggest that cultivar-specific salt tolerance may vary across developmental stages, such that a genotype tolerant at one stage may not exhibit tolerance at another (Satasiya et al., 2024). This stage-dependent variability can introduce inconsistencies across datasets, indicating that more accurate results may be achieved through comprehensive data integration and overall enrichment analysis. Such approaches can improve data accuracy and highlight the need for greater precision in QTL mapping (Sharma et al., 2023).

Meta-analysis, overview analysis, and collinearity analysis can each be used to integrate results from previous studies. Accurate localization of QTLs is an important prerequisite for fine gene mapping and gene cloning, as these approaches integrate a large number of scattered QTLs, shorten confidence intervals, facilitate the identification of major-effect loci, improve the accuracy and effectiveness of QTL mapping, and reduce false-positive QTLs generated during QTL mapping (Raj and Nadarajah, 2022). In the present study, 265 QTL related to salt tolerance were collected, which were sufficient to reliably locate candidate regions. Overview analysis, collinearity analysis, and meta-analysis were performed to narrow the interval. The results showed that the target region was concentrated on chromosome 9, and the interval was reduced from 3.5 to 0.36 Mb. Similarly, meta-analysis, overview analysis, and collinearity analysis have been used to identify candidate regions in other crops such as wheat (Sharma et al., 2023), pea (Hamon et al., 2013), and grapevine (Delfino et al., 2019).

### 4.2 Expression analysis and verification of OsMOCSalt

Functional annotation of genes within the candidate region is an important approach for mining genes, especially after QTL analysis (Raj and Nadarajah, 2022). Several functional genes have been identified and screened through such functional annotation (Selamat and Nadarajah, 2021). In the present study, 36 genes were annotated in the region, which may be closely related to salt tolerance, and the results are consistent with findings reported in wheat (Guo et al., 2022) and upland cotton (Guo et al., 2021). Additionally, gene expression levels may provide further insights into gene function, and the Phytozome database provides expression data (Zhang et al., 2022a). Genes with higher expression levels may be more functionally relevant than those with lower levels, suggesting that screening genes based on expression levels is an effective strategy (Zhang et al., 2025). For example, *Os06g10650* and *Os06g10710* showed significant differential transcriptional abundance among five candidate genes in qGR6.2 (a QTL in rice), and *Os06g10650*, which was identified through expression-based screening, was upregulated under salt stress in both parents (Zeng et al., 2021).

In the present study, four genes were screened through expression analysis, and only *OsMOCSalt* exhibited significant differences in expression levels between control and salt stress conditions. The expression levels of other genes, compared between control and salt stress conditions, did not show consistent significant differences across all cultivars (**Figure 5**), thereby further narrowing the number of candidate genes. Moreover, *Os09g0416200* (*OsMOCSalt*) encodes a monosaccharide transporter exclusively localized to the rice Golgi apparatus, which executes unique sugar translocation functions separate from plasma membrane and tonoplast sugar transporters characterized in salt response research. Transcript analysis demonstrated that salt stress significantly induced *Os09g0416200* expression, indicative of its active participation in adaptive responses to high salinity in rice, such that attenuated *Os09g0416200* expression disturbed intracellular monosaccharide distribution and depleted osmoprotective soluble sugar reserves, ultimately resulting in exacerbated salt-induced growth inhibition in rice (Cao et al., 2011), which further supports the reliability of the present study.

Genes undergo mutations and form different haplotypes during evolution, and different haplotypes can directly affect gene expression levels and crop phenotypes (Habila et al., 2022). In this study, SNP_1_ and SNP_2_ of *OsMOCSalt* were analyzed; only SNP_1_ showed phenotypic differences in salt tolerance, suggesting that SNP_1_ may represent a functional mutation site. Furthermore, yeast transformation provides a fundamental approach to preliminarily verify gene function, as yeast is one of the simplest eukaryotic organisms and has been widely utilized as an effective validation system (Zhang et al., 2025). Although yeast heterologous expression systems serve as a rapid and efficient tool for preliminary functional verification of candidate salt tolerance genes, this approach possesses inherent limitations that cannot be ignored. The function of target genes and salt tolerance regulatory characteristics identified via yeast validation only reflect the basic molecular function of target genes in heterologous systems, two *OsMOCSalt* haplotypes were expressed in transgenic yeast, and *OsMOCSalt^Hap1^* exhibited greater salt tolerance than *OsMOCSalt^Hap2^*, indicating that *OsMOCSalt* functions as a salt tolerance-related gene and that *OsMOCSalt^Hap1^* is a favorable salt-tolerant haplotype. Although yeast-based verification is still preliminary and may not fully reflect gene function across different species (Zhang et al., 2025), the observed spatiotemporal expression pattern and regulatory mechanism of *OsMOCSalt^Hap1^* in rice should be further validated in future studies.

**Figure 8:**
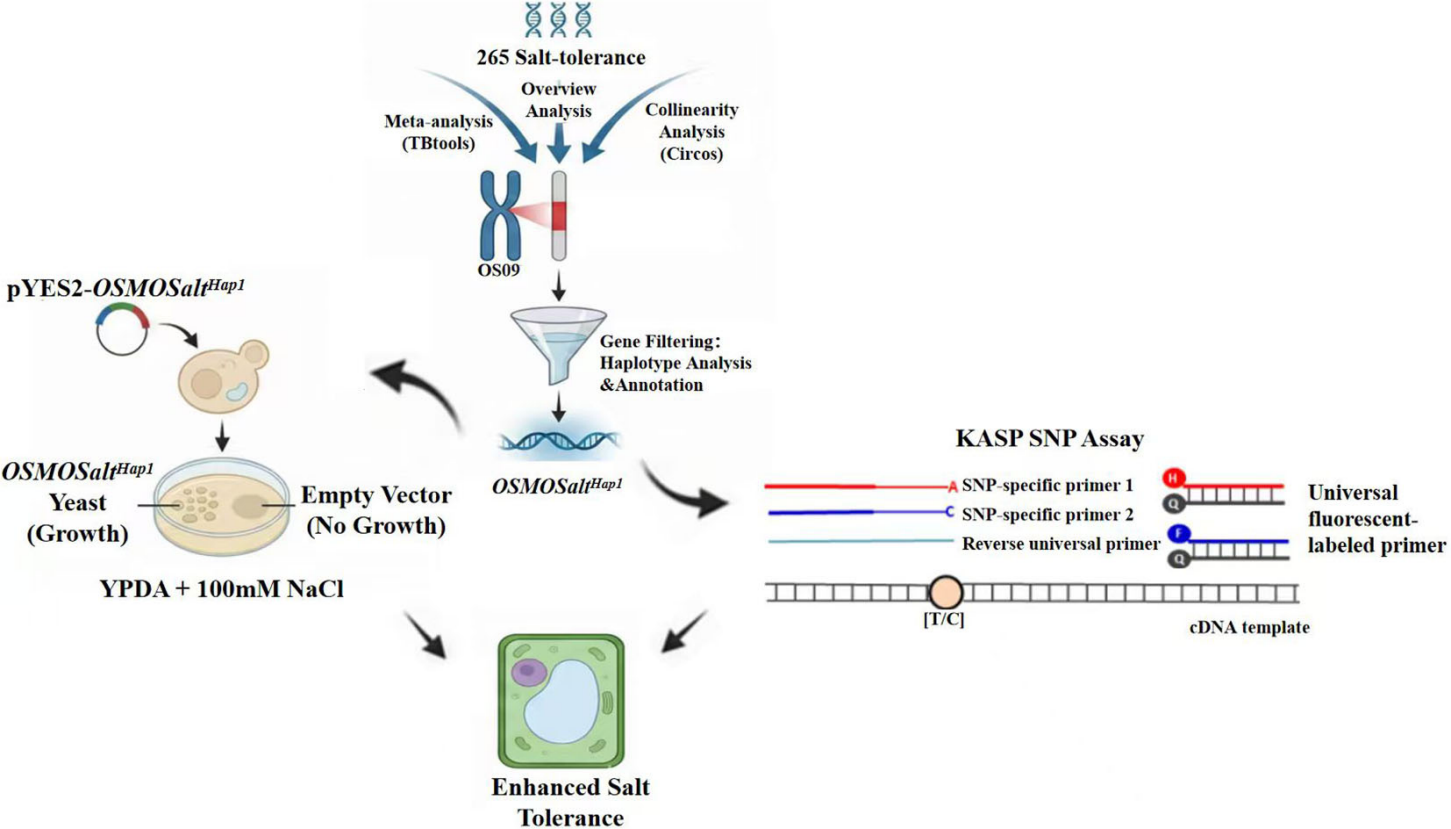
Identification, analysis, and validation of *OsMOCSalt* in rice (*Oryza sativa*). *OsMOCSalt^Hap1^* was identified as the salt-tolerant haplotype, as validated by transgenic yeast assays and KASP molecular marker analysis.

Molecular breeding, especially through targeted genetic engineering of candidate genes and the development of transgenic lines, can utilize QTLs to improve crop performance. Identification of candidate genes is essential for understanding the molecular and physiological mechanisms underlying drought tolerance in crops (Vikram et al., 2011). This information can be applied not only in transgenic research but also in the development of molecular markers for selection and screening (Liang et al., 2024). For example, a specific RAPD marker tightly linked to a salt tolerance gene has been identified and used for screening salt-tolerant accessions, and a sequence-characterized amplified region marker has been developed for fine mapping of salt tolerance genes (Guan et al., 2014). The use of molecular markers in marker-assisted breeding dramatically improves breeding efficiency by enabling direct genotype selection and represents a core technology used in modern precision breeding. In the present study, KASP primers for *OsMOCSalt^Hap1^* were developed and analyzed, with key implications for salt tolerance breeding in rice (**Figure 8**).

## 5 Conclusion

In this study, a key candidate genomic region was identified through overview analysis, collinearity analysis, and meta-analysis on chromosome 9 within the 14.85-15.21 Mb region. A total of 36 genes were identified in this region and subsequently screened through gene annotation and expression analysis using the Phytozome database. A potential candidate gene (*OsMOCSalt*) was identified, and its SNPs were analyzed. *OsMOCSalt^Hap1^* was identified as a salt-tolerant allele based on transgenic yeast and KASP molecular marker analyses, suggesting its potential utility in breeding programs. This study provides candidate genes, valuable information, and an empirical basis for breeding, especially for salt-tolerant rice breeding.

## Data Availability

The original contributions presented in the study are included in the article/**Supplementary Material**. Further inquiries can be directed to the corresponding authors.
