# Supplementary material for: OsMOCSalt as a candidate salt tolerance gene in rice (Oryza sativa) identified through meta-analysis, overview analysis and collinearity analysis
Source: Front Plant Sci. 2026 Jul 24;17:1868272. doi: 10.3389/fpls.2026.1868272 (PMC13449998; doi:10.3389/fpls.2026.1868272)
Supplement: Supplementary file 1 [file Table2.docx]

Table S2: The germplasm resources and salt tolerance identification results of rice in this study

| **Variety** | **Salt tolerance** |
| --- | --- |
| Extra Long Grain Fragrant | 1 |
| Green Pearl 2 | 1 |
| Harbin Long Breeding 1 | 1 |
| Harbin Agricultural Breeding 1 | 1 |
| Song Sticky 1 | 1 |
| Tai Fragrant King | 1 |
| Long Rice 28 | 1 |
| Zhonglong Japonica 3 | 1 |
| Ken Rice 12 | 1 |
| Long Japonica 25 | 1 |
| Xingfu 1 | 2 |
| Zhongke Rice 32 | 2 |
| Zhongke 613 | 2 |
| Wokeshou 1 | 2 |
| Dongfu 101 | 2 |
| Dongfu 105 | 2 |
| Dongfu 117 | 2 |
| Harbin Japonica Rice 2 | 2 |
| Ken Rice 15 | 2 |
| Long Rice 16 | 2 |
| Zhonglong Japonica 15 | 2 |
| Long Rice 22 | 2 |
| Ken Rice 10 | 2 |
| Long Japonica 14 | 2 |
| Songhua Japonica 10 | 2 |
| Long Rice 15 | 2 |
| Long Rice 3 | 2 |
| Sui Japonica 7 | 2 |
| Long Japonica 42 | 2 |
| Jilin Japonica 815 | 2 |
| Jilin Japonica 526 | 2 |
| Shenyang Rice 27 | 2 |
| Beijing Japonica 3 | 4 |
| Dongfu 125 | 4 |
| Changbai 15 | 4 |
| Golden Grain 1 | 4 |
| Songhua Japonica 3 | 4 |
| Fragrant Sticky King | 4 |
| Long Japonica 40 | 4 |
| Songhua Japonica 12 | 4 |
| Tong Breeding 308 | 4 |
| Songhua Japonica 29 | 4 |
| Wuyou Rice 1 | 4 |
| Dongnong 415 | 4 |
| Daole Fragrant | 4 |
| Fangyou 4 | 4 |
| Longqing Rice 30 | 4 |
| Sui Japonica 11 | 4 |
| Long Rice 8 | 4 |
| Longdao 203 | 5 |
| Songhua Japonica 2 | 5 |
| Jilin 7 | 5 |
| Longdao 209 | 5 |
| Long Rice 14 | 5 |
| Longdao 133 | 5 |
| Songhua Japonica 1 | 5 |
| Dongdao 12 | 5 |
| Songhua Japonica 5 | 5 |
| Songhua Japonica 7 | 5 |
| Sui Japonica 13 | 5 |
| Songhua Japonica 18 | 5 |
| Long Fragrant Glutinous | 5 |
| Dalin 1 | 5 |
| Mudanjiang 27 | 5 |
| Mudanjiang 31 | 5 |
| Songhua Japonica 19 | 5 |
| Songhua Japonica 57 | 5 |
| Song 12 | 5 |
| Zhonglong Fragrant Japonica 1 | 5 |
| Mudanjiang 32 | 5 |
| Weinong 301 | 5 |
| Fragrant Rice | 5 |
| Daohua Fragrant | 5 |

**Note: Number from 1 to 5 represent increasing salt tolerance.**
