# Supplementary material for: OsMOCSalt as a candidate salt tolerance gene in rice (Oryza sativa) identified through meta-analysis, overview analysis and collinearity analysis
Source: Front Plant Sci. 2026 Jul 24;17:1868272. doi: 10.3389/fpls.2026.1868272 (PMC13449998; doi:10.3389/fpls.2026.1868272)
Supplement: Supplementary file 3 [file Table4.docx]

Table S4: The RT-qPCR primers under control and salt stress used in the study;

| Primer | Sequence（5’-3’） |
| --- | --- |
| *Os09g0416200-F* | ACTACGGGACGCAGCACA |
| *Os09g0416200-R* | CAGGCTGTTGGGCGTCT |
| *Os09g0416800-F* | TTTCAACTACGCAACGCT |
| *Os09g0416800-R* | GAACTGCCAAACACAAGG |
| *Os09g0419200-F* | ACCCGAAGAACGCCCA |
| *Os09g0419200-R* | TACTCCGTCCCCCGCA |
| *Os09g0420800-F* | GCCGACTACAACATTC |
| *Os09g0420800-R* | CCTTCTTTCTCCTCAA |
| *Osactin-F* | TACAGTGTCTGGATTGGAGGA |
| *Osactin-R* | TCTTGGCTTAGCATTCTTGGGT |
