# Supplementary material for: OsMOCSalt as a candidate salt tolerance gene in rice (Oryza sativa) identified through meta-analysis, overview analysis and collinearity analysis
Source: Front Plant Sci. 2026 Jul 24;17:1868272. doi: 10.3389/fpls.2026.1868272 (PMC13449998; doi:10.3389/fpls.2026.1868272)
Supplement: Supplementary file 4 [file Table5.docx]

Table S5: The primers of *OsMOCSalt* in transgenic yeast;

| Primer | Sequence（5’-3’） |
| --- | --- |
| P-YES-F | AGGGAATATTAAGCTATGGCGGGAGGCGGC |
| P-YES-R | GATATCTGCAGAATTCTTGTGATGACTGTCGCCGT |
