# Supplementary material for: OsMOCSalt as a candidate salt tolerance gene in rice (Oryza sativa) identified through meta-analysis, overview analysis and collinearity analysis
Source: Front Plant Sci. 2026 Jul 24;17:1868272. doi: 10.3389/fpls.2026.1868272 (PMC13449998; doi:10.3389/fpls.2026.1868272)
Supplement: Supplementary file 5 [file Table6.docx]

Table S6: The KASP primers of SNP_1_ sites in this study;

| Primer | Sequence（5’-3’） |
| --- | --- |
| SNP_1_-F_1_ | GAAGGTGACCAAGTTCATGCTCGCCACGCTCAACGCCGCCGCCGTT |
| SNP_1_-F_2_ | GAAGGTCGGAGTCAACGGATTCGCCACGCTCAACGCCGCCGCCGTC |
| SNP_1_-R | AGCAGGATGCGGCCGAGGATGAGCATGAC |
| SNP_2_-F_1_ | GAAGGTGACCAAGTTCATGCTTGTCTGAATCCTCGTGAAAATATTA |
| SNP_2_-F_2_ | GAAGGTCGGAGTCAACGGATTTGTCTGAATCCTCGTGAAAATATTG |
| SNP_2_-R | GTAAAGTTTATTTTTGCTACAATCTGAAC |
