# Supplementary material for: OsMOCSalt as a candidate salt tolerance gene in rice (Oryza sativa) identified through meta-analysis, overview analysis and collinearity analysis
Source: Front Plant Sci. 2026 Jul 24;17:1868272. doi: 10.3389/fpls.2026.1868272 (PMC13449998; doi:10.3389/fpls.2026.1868272)
Supplement: Supplementary file 6 [file Table7.docx]

Table S7 : The genes’ function in the candidate region through overview-, collinear-, and meta- analysis.

| Gene ID | Function |
| --- | --- |
| *Os09g0415700* | Encodes a putative protein, it is a predicted gene with low expression in the rice genome. |
| *Os09g0415750* | Encodes a putative protein containing leucine-rich repeat (LRR) domain; LRR domains are usually involved in protein-protein interactions and signal transduction. |
| *Os09g0415800* | Encodes a putative transmembrane protein, predicted to be involved in material transport or signal transmission on the cell membrane. |
| *Os09g0416200* | Encodes glutathione S-transferase. |
| *Os09g0416250* | Encodes a putative ubiquitin-conjugating enzyme E2-like protein; ubiquitin-conjugating enzymes are involved in the ubiquitination modification process, and this gene may participate in the regulation of protein degradation in rice. |
| *Os09g0416500* | Encodes a putative serine/threonine protein kinas. |
| *Os09g0416501* | Encoding a truncated putative serine/threonine protein kinase with no independently verified biological function. |
| *Os09g0416600* | Encodes a putative nucleic acid-binding protein, predicted to be involved in gene transcription regulation, expressed at trace levels in rice root tissues. |
| *Os09g0416700* | Encodes a putative hydrolase belonging to the α/β hydrolase superfamily, predicted to be involved in the hydrolytic metabolism of organic molecules. |
| *Os09g0416800* | Encodes a putative heat shock protein 70 (HSP70)-like protein; heat shock proteins are usually involved in cellular stress protection. |
| *Os09g0416900* | Encodes a putative ribosomal protein L23a; ribosomal proteins are involved in protein biosynthesis, and this gene is constitutively expressed at low levels in various rice tissues. |
| *Os09g0417400* | Encodes a putative member of the ABC transporter protein family,involved in the transmembrane transport of various substances, and this gene is expressed in small amounts in rice stems. |
| *Os09g0417500* | Encodes a putative metal ion-binding protein, predicted to be involved in the homeostasis regulation of metal ions in rice. |
| *Os09g0417600* | Encodes a putative lipoxygenase-like protein related to jasmonic acid synthesis; jasmonic acid is involved in plant defense responses. |
| *Os09g0417800* | Encodes a putative transcription factor containing MYB domain; MYB transcription factors are involved in plant development and stress responses. |
| *Os09g0418000* | Encodes a putative cytochrome P450 enzyme (CYP450), predicted to be involved in rice secondary metabolism (e.g., phytoalexin synthesis, corresponding to GO Biological Process/BP: secondary metabolite biosynthetic process), with cellular localization predicted to be endoplasmic reticulum (Cellular Component/CC: endoplasmic reticulum membrane) and possessing monooxygenase activity (Molecular Function/MF: heme binding, monooxygenase activity). |
| *Os09g0418250* | Encodes a putative leucine-rich repeat (LRR) receptor-like protein kinase, predicted to participate in plant signal transduction (BP: cellular signal transduction), mediate protein-protein interaction via LRR domain (MF: protein binding), and localize to cell membrane (CC: plasma membrane); may be related to stress response or growth regulation. |
| *Os09g0418375* | Encodes a putative ubiquitin ligase E3 (RING domain protein), predicted to be involved in protein ubiquitination (BP: protein ubiquitination), with ubiquitin transferase activity (MF: RING-type E3 ubiquitin ligase activity), and localize to cytoplasm (CC: cytoplasm); may regulate target protein degradation (e.g., stress-related proteins). |
| *Os09g0418500* | Encodes a putative ABC transporter (ABCG subfamily), predicted to participate in transmembrane substance transport (BP: small molecule transmembrane transport), with ATP binding and hydrolysis activity (MF: ATP binding, ATPase activity), and localize to cell membrane (CC: plasma membrane); may be involved in efflux of secondary metabolites or toxins. |
| *Os09g0418800* | Encodes a putative heat shock protein 90 (HSP90), predicted to be involved in protein folding and stress protection (BP: protein homeostasis maintenance, heat stress response), with chaperone activity (MF: protein binding, chaperone activity), and localize to cytoplasm and endoplasmic reticulum (CC: cytoplasm, endoplasmic reticulum); may assist correct folding of target proteins under high temperature stress. |
| *Os09g0418700* | Encodes a putative sucrose synthase, predicted to participate in carbohydrate metabolism (BP: sucrose metabolism, glycolytic precursor generation), with sucrose cleavage activity (MF: sucrose synthase activity, UDP-glucose binding activity), and localize to cytoplasm (CC: cytoplasm); may regulate sucrose-to-starch conversion in rice grains. |
| *Os09g0419100* | Encodes a putative MYB transcription factor (R2R3-type), predicted to participate in gene transcription regulation (BP: transcriptional regulation, plant organ development), with DNA binding activity (MF: specific DNA sequence binding, transcription factor activity), and localize to nucleus (CC: nucleus); may be involved in leaf senescence or floral organ development regulation. |
| *Os09g0419200* | Encodes a putative phospholipase C (PLC), predicted to participate in phospholipid signaling pathway (BP: phospholipid metabolism, intracellular signal cascade), with phospholipase activity (MF: phospholipase C activity, inositol trisphosphate-generating activity), and localize to inner side of plasma membrane (CC: inner plasma membrane); may play a role in stress signal (e.g., salt stress) transmission. |
| *Os09g0419500* | Encodes a putative metallothionein (MT), predicted to participate in metal ion homeostasis (BP: heavy metal ion detoxification, metal ion transport), with metal ion binding activity (MF: cadmium ion binding, zinc ion binding), and localize to cytoplasm (CC: cytoplasm); may enhance rice tolerance to heavy metal stress. |
| *Os09g0419600* | Encodes a putative ribonuclease H (RNase H), predicted to participate in nucleic acid metabolism (BP: RNA degradation, retrotranscribed RNA clearance), with ribonuclease activity (MF: ribonuclease H activity, RNA binding), and localize to nucleus and mitochondria (CC: nucleus, mitochondrial matrix); may be involved in viral RNA defense or genome stability maintenance. |
| *Os09g0420000* | Encodes a putative proline dehydrogenase (ProDH), predicted to participate in amino acid metabolism (BP: proline catabolism, oxidative stress response), with dehydrogenase activity (MF: proline dehydrogenase activity, FAD binding), and localize to mitochondria (CC: mitochondrial membrane); may regulate metabolic flux in proline-mediated stress tolerance. |
| *Os09g0420100* | Encodes a putative calmodulin-binding protein (CaMBP), predicted to participate in calcium signaling pathway (BP: calcium-mediated signal transduction, cell cycle regulation), with calmodulin binding activity (MF: calmodulin binding, calcium ion sensing), and localize to cytoplasm and nucleus (CC: cytoplasm, nucleus); may be associated with signal integration of growth and stress response. |
| *Os09g0420200* | Encodes a putative pectin methylesterase (PME), predicted to participate in cell wall metabolism (BP: cell wall modification, cell elongation), with pectin methylesterase activity (MF: pectin methylesterase activity, carbohydrate esterase activity), and localize to cell wall (CC: cell wall); may regulate rice stem strength or fruit development. |
| *Os09g0420300* | Encodes a putative glutathione reductase (GR), predicted to participate in redox homeostasis (BP: glutathione metabolism, antioxidant defense), with reductase activity (MF: glutathione reductase activity, NADPH binding), and localize to chloroplasts and mitochondria (CC: chloroplast stroma, mitochondrial matrix); may play a role in light stress or oxidative damage repair. |
| *Os09g0420600* | Encodes a putative transporter (MATE family), predicted to participate in secondary metabolite transport (BP: alkaloid transport, organic acid secretion), with drug/metabolite transport activity (MF: organic cation transport activity, H⁺-dependent transport activity), and localize to cell membrane (CC: plasma membrane); may be involved in extracellular secretion of rice stress-related metabolites. |
| *Os09g0420700* | Encodes a putative serine carboxypeptidase (SCP), predicted to participate in protein processing (BP: peptide chain degradation, protein maturation), with carboxypeptidase activity (MF: serine-type carboxypeptidase activity, peptide bond hydrolase activity), and localize to vacuole (CC: vacuolar lumen); may play a role in protein degradation during seed germination. |
| *Os09g0420800* | Encodes a putative cyclic nucleotide-gated channel protein (CNGC), predicted to participate in ion transport (BP: calcium ion transmembrane transport, stress signal transduction), with ion channel activity (MF: cyclic nucleotide-gated ion channel activity, calcium ion channel activity), and localize to cell membrane (CC: plasma membrane); may play a role in ion signaling of salt stress or pathogen defense. |
| *Os09g0420900* | Encodes a putative histone deacetylase (HDAC), predicted to participate in epigenetic regulation (BP: chromatin modification, gene silencing), with histone deacetylase activity (MF: histone deacetylase activity, zinc ion binding), and localize to nucleus (CC: chromatin); may regulate gene expression patterns during rice development stages. |
| *Os09g0421100* | Encodes a putative receptor-like protein (RLP, no kinase domain), predicted to participate in cell recognition (BP: pathogen-associated molecular pattern recognition, immune response), with protein binding activity (MF: extracellular ligand binding, receptor activity), and localize to cell membrane (CC: outer plasma membrane); may be involved in rice disease resistance defense response. |
| *Os09g0421133* | An alternative splicing variant of Os09g0421100, encoding a truncated receptor-like protein (lacking part of extracellular domain), predicted to retain partial protein binding ability (MF: partial ligand binding activity), with possible cellular localization to endoplasmic reticulum (CC: endoplasmic reticulum); no independent biological function verification, speculated to assist in processing or transport of the full-length protein. |
| *Os09g0421166* | Another alternative splicing variant of Os09g0421100, encoding a short peptide without extracellular domain, predicted to have no complete receptor function (MF: no clear molecular activity), and localize to cytoplasm (CC: cytoplasm); may be a non-functional transcript or involved in protein degradation regulation. |
